# Revisiting the Diagnostic Performance of the Modified Nine-Step Test for Obstructive and Patulous Eustachian Tube Dysfunction

**DOI:** 10.3390/diagnostics12030732

**Published:** 2022-03-17

**Authors:** Seong Hoon Bae, Seojin Moon, Mincheol Jeong, In Seok Moon

**Affiliations:** Department of Otorhinolaryngology, Yonsei University College of Medicine, Seoul 03722, Korea; bshsap@naver.com (S.H.B.); jinmoon31@yuhs.ac (S.M.); jmc8237@yuhs.ac (M.J.)

**Keywords:** nine-step test, eustachian tube, eustachian tube dysfunction, patulous eustachian tube

## Abstract

The nine-step test is a classical method for evaluating Eustachian tube function. It directly assesses the patient’s capacity to equilibrate middle ear pressure by swallowing. However, there are insufficient studies to appraise its diagnostic performance. The purpose of this study is to evaluate the sensitivity, specificity, and cut-off value of the nine-step test in patients with obstructive Eustachian tube dysfunction (oETD) and patulous Eustachian tube (PET). Enrolled subjects were divided into three groups. Control (50 ears of healthy volunteers), oETD (19 ears with oETD), and PET (29 ears with PET). Receiver operating characteristics curve analysis was conducted to evaluate the diagnostic performance of maximal peak pressure difference (ETTmd) in the nine-step test. Both the oETD group and the PET group showed decreased ETTmd. The nine-step test showed moderate accuracy when used to diagnose oETD (area under the curve = 0.875) and PET (area under the curve = 0.769). The highest diagnostic performance was observed when the cut-off value was 13 daPa for both the oETD group (sensitivity = 73.7%, specificity = 90.0%) and the PET group (sensitivity = 58.6%, specificity = 90.0%). The nine-step test has moderate diagnostic performance for oETD and PET.

## 1. Introduction

Eustachian tube dysfunction (ETD) is classified into three categories namely, obstructive (oETD), barochallenging (bETD), and patulous (PET) [1]. Patients with oETD complain of characteristic symptoms of ear fullness, muffled hearing, and other non-specific audiological symptoms. Those with bETD suffer from severe otalgia from changes in ambient pressure, typically in airplanes or diving. Finally, PET is associated with symptoms such as autophony, breathing sounds in ear, and fluttering of the tympanic membrane during respiration [1,2].

There is currently no gold standard method for evaluating Eustachian tube function so far. Previous studies have used negative middle ear pressure or tympanic membrane abnormalities (e.g., fluid collection in the middle ear, retracted drum) as objective findings for diagnosing oETD [1,3,4]. The nine-step test directly assesses a patient’s ability to equilibrate middle ear pressure by swallowing, but its diagnostic value has not been sufficiently appraised. However, the nine-step test is inapplicable to abnormal tympanic membranes, and thus has limited clinical value to diagnose oETD in these cases. Nevertheless, it may still be used in cases of oETD where the tympanic membrane is normal. The nine-step test may also have a role in the diagnosis of bETD because the mechanism of the nine-step test is similar to its pathophysiology [5,6,7,8]. In addition, PET may also be diagnosed using the nine-step test because the patulous Eustachian tube fails to maintain middle ear pressure following the change in external auditory canal (EAC) air pressure.

Despite its advantages, there is still insufficient data to validate the use of the nine-step test in oETD and PET. Furthermore, the reference maximal peak pressure difference (ETTmd), which reflects healthy Eustachian tube function in the nine-step test, also varies from study to study [4,7,8]. Therefore, the purpose of this study is to evaluate the diagnostic value of the nine-step test in oETD and PET patients. We recruited healthy volunteers to evaluate the cut-off values of ETTmd using receiver operating characteristics (ROC) curve analysis. Herein, the sensitivity and specificity of the nine-step test using different cut-off values for diagnosing oETD and PET will be discussed.

## 2. Materials and Methods

### 2.1. Subject Enrollment

The subjects were enrolled and categorized into three groups. The control groups consisted of 50 ears from 25 healthy volunteers with no history of otologic disease and no experience of pain on flights or when diving. The age of healthy volunteers ranged from 20 to 39 years old.

The patient groups were retrospectively enrolled. These groups included patients suspected of ETD who visited our clinic from 5 May 2021 to 5 January 2022. The oETD group consisted of 19 ears from 14 patients. Additional inclusion criteria for the oETD group were (1) presentation of typical symptoms; and (2) middle ear pressure below −50 daPa or retracted tympanic membrane [9,10,11]. The exclusion criteria were (1) perforated tympanic membrane; (2) otitis media; and (3) not having a distinctive peak in impedance audiometry (e.g., type B). The PET group consisted of 29 ears from 17 patients. The inclusion criteria for PET were based on the Japan Otological Society guidelines (possible and definite PET) [2]. These were (1) presence of typical symptoms such as ear fullness, autophony, and breathing sound in the ear; (2) relief of symptoms by positional changes such as lying or bending; and (3) drum flattering synchronized with breathing, confirmed by oto-endoscopy. This study was approved by the institutional review board of the author’s affiliated hospital (4-2021-1180).

### 2.2. Eustachian Tube Function Evaluation

All enrolled subjects answered a Korean version of the ETDQ-7 survey. The survey consisted of seven questionnaires regarding representative symptoms of ETD [12]. The participants also underwent otoscopic examination immediately followed by a Eustachian tube function test using GSI TympStar Pro (Grason-Stadler Inc., Eden Prairie, MN, USA). The Eustachian tube function test was based on Bluestone’s nine-step test. Evaluation of middle ear pressure was done in a normal state, followed by the introduction of 400 daPa of negative pressure into the ear canal. Participants were then asked to dry swallow thrice before reevaluation of middle ear pressure. The same procedure was then performed using 400 daPa of positive pressure. The maximal difference in the middle ear pressure from the three states reflects Eustachian tube function. Because a standard reference range of ETTmd has been not established, we also analyzed the cut-off values of 10 daPa and 15 daPa, based on previous studies that used a nine-step test as a diagnostic tool for ETD [3,4,7,8].

### 2.3. Statistical Analyses

For continuous variables, the Kruskal–Wallis test was conducted with post hoc Dunn’s multiple comparisons test to compare the results of three groups. In the case of proportional values, Fisher’s exact test was used to evaluate statistical significance. The ROC curve was visualized using Prism 8.0 (GraphPad Software, San Diego, CA, USA). The sensitivity and specificity were evaluated from the ROC curve. All statistical analyses were conducted using IBM SPSS version 20 (IBM Co., Armonk, NY, USA), and *p* < 0.05 was considered statistically significant.

## 3. Results

### 3.1. Distribution of Nine-Step Test Results in the Three Groups

**Figure 1:**
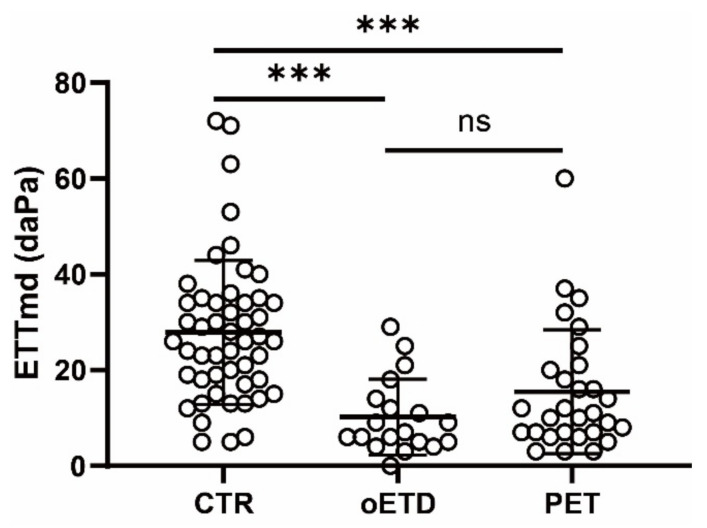
Eustachian tube function of the three groups. ETTmd: maximal difference of peak pressure in the nine-step test, CTR: control group, oETD: obstructive Eustachian tube dysfunction group, PET: patulous Eustachian tube dysfunction group. ***: *p* < 0.001. ns: *p* > 0.05.

The ETTmd values were significantly lower in the oETD (10.2 ± 8.4, *p* < 0.001) and PET (15.5 ± 12.9, *p* < 0.001) groups than in the control group (27.9 ± 15.0) (Table 1 and Figure 1). However, the difference in ETTmd between the oETD group and the PET group was insignificant (*p* = 0.474). The mean middle ear pressure was significantly negative in the oETD group (−152.1 ± 113.4) compared to those in the control group (−9.4 ± 11.5, *p* < 0.001) and the PET group (−10.3 ± 21.8, *p* < 0.001). However, the mean middle ear pressures of the control group and the PET group were similar (*p* = 1.0). Taken together, the ETTmd was decreased in both oETD and PET groups; however, middle ear pressure was abnormal only in the oETD group.

### 3.2. Clinical Value for Diagnosing oETD in the Nine-Step Test

**Figure 2:**
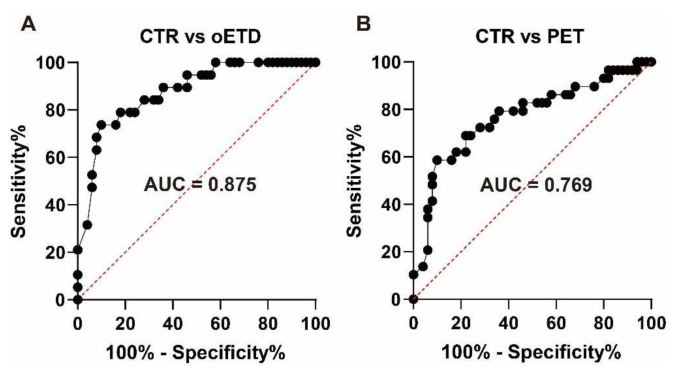
Receiver operating characteristics curve analysis. (**A**) For diagnosing oETD, (**B**) for diagnosing PET. AUC: Area under the curve, CTR: Control group, oETD: obstructive Eustachian tube dysfunction group, PET: patulous Eustachian tube dysfunction group.

We evaluated the clinical value of the nine-step test in the diagnosis of oETD using ROC curve analysis (Figure 2A). The area under the curve was 0.875 (*p* < 0.001), which indicated moderate accuracy (Table 2) [13]. The ETTmd cut-off value of 13 daPa showed the highest diagnostic performance, with a sensitivity of 73.7% and a specificity of 90.0%. The sensitivity and specificity were 79.0% and 82.0%, respectively, when the cut-off value was 15 daPa. The sensitivity and specificity were 63.2% and 92.0%, respectively, when the cut-off value was 10 daPa.

### 3.3. Clinical Value for Diagnosing PET in the Nine-Step Test

We also evaluated the clinical value of the nine-step test in the diagnosis of PET using ROC curve analysis (Figure 2B). The area the under curve was 0.769 (*p* < 0.001), which indicated moderate accuracy (Table 2). The ETTmd cut-off value of 13 daPa showed the highest diagnostic performance, with a sensitivity of 58.6% and a specificity of 90.0%. The sensitivity and specificity were 62.1% and 82.0%, respectively, when the cut-off value was 15 daPa. The sensitivity and specificity were 48.3% and 92.0%, respectively, when the cut-off value was 10 daPa.

## 4. Discussion

The nine-step test showed moderate accuracy when used to diagnose oETD (area under the curve = 0.875) and PET (area under the curve = 0.769) in this study. The highest diagnostic performance was observed with a set ETTmd cut-off value of 13 daPa for both oETD (sensitivity = 73.7%, specificity = 90.0%) and PET (sensitivity = 58.6%, specificity = 90.0%).

Both the oETD group and the PET group had decreased ETTmd. However, the PET group showed normal middle ear pressure similar to the control group. The decreased ETTmd in the PET group is likely attributable to pseudo-dysfunction, which occurs due to the failure to maintain the pressure in the middle ear. The equilibrated air should be trapped in the middle ear following deglutition since the Eustachian tube closes in a resting state. However, in the PET patients, the trapped air leaks through the patulous Eustachian tube. Thus, middle ear pressure equilibrates to nasopharyngeal pressure regardless of EAC pressure changes. Consequently, the PET group can exhibit decreased ETTmd with normal middle ear pressures. In addition to thorough history taking and physical examination, the middle ear pressure is important for differential diagnosis between PET and oETD, as the result of the nine-step test in PET mimicking oETD.

Ambient pressure tympanometry is another diagnostic tool used for PET patients. It can objectively show respiration-synchronous wave patterns and has been reported to have 53.3% sensitivity and 93.9% specificity [14,15]. This is comparable to the diagnostic performance of the nine-step test in this study. However, the intermittent character of PET symptoms may affect the sensitivity of both tests. The differences in the diameters of the patent Eustachian tube in each patient can also lead to varying spontaneous air ventilation. These variations may also affect results and explain the low sensitivity of ETTmd. Nevertheless, ETTmd may still be considered as an additional diagnostic test for PET.

There is currently no gold standard test for oETD, making it more challenging to diagnose compared with PET. Sonotubometry and tubomanometry are widely accepted quantitative Eustachian tube tests. They have up to 90% and 93% specificity, respectively [16,17]. However, their sensitivity for oETD has not been clearly established, possibly due to a lack of standard diagnostic criteria. In the case of serous otitis media, Eustachian tube opening measured by sonotubometry and tubomanometry has a sensitivity of 47% and 49%, respectively [17,18]. Because sonotubometry, tubomanometry, and the nine-step test all evaluate the opening of the Eustachian tube, their diagnostic performance may be similar. However, the nine-step test had a higher sensitivity in this study. This may have resulted from the subject selection process. One of the inclusion criteria for this study is the middle ear pressure of −50 daPa and below. The abnormal middle ear pressure (type C tympanogram) is generally defined as below −100 daPa [19,20]. However, other studies have also defined abnormal middle ear pressure as below −50 daPa [9,10,11,17,21]. More recently, a prospective study by Parsel et al. suggested a cut-off value for abnormal middle ear pressure to be between 25–50 daPa [22]. Our study also excluded patients with perforations or otitis media. This was done because a crucial disadvantage of the nine-step test is its applicability only to normal tympanic membranes. It cannot be used in patients with perforations or middle ear fluid effusion. oETD can theoretically cause serous otitis media or adhesive otitis media, making the test inapplicable to a significant proportion of patients [23]. Conversely, the nine-step test can measure middle ear pressure in a single test. This may be beneficial for diagnosing oETD and oETD related complications.

The cut-off value of the nine-step test varies from study to study, ranging from 10 to 15 daPa. The cut-off initially suggested by the manufacturer is 15 daPa; however, this had not been validated. In a study by Hussein et al., a cut-off of 10 daPa yielded a sensitivity of 91% and a specificity of 100% when diagnosing bETD [6]. The high sensitivity and specificity of the test for bETD may be due to the similarity of the test mechanism and disease pathophysiology. Indeed, the nine-step test showed good performance compared to the other Eustachian tube function tests [5].

Although rarely done due to its problems with applicability, the nine-step test has also been used to evaluate oETD. However, there is insufficient evidence to assess its diagnostic performance in this regard. In previous studies, cut-off values also ranged from 10 daPa to 15 daPa, depending on the study protocol [3,4,7,8]. The cut-off value that showed the best performance in this study was 13 daPa. Clinically, oETD may not be limited to patients who have severely negative middle ear pressure because Eustachian tube ballooning is also effective for patients with oETD symptoms and normal middle ear pressure [10,22,24,25]. Therefore, the result of this study can be applied to oETD patients with normal middle ear pressure.

As a limitation, it is important to note that the device (GSI TympStar Pro) used in this study was a modified version of the nine-step test, originally conceptualized by Bluestone in 1975 [26]. In the original methodology, impedance audiograms were measured five times. However, GSI TympStar Pro only measured audiograms three times. The device also omits the test of equilibrated to ambient pressure status following the deflation/inflation test. Given that the value indicating Eustachian tube function is the maximal difference in middle ear pressure, this omission may not largely affect the results. However, it is still necessary to validate the modified methodology and compare it with the original technique. Another limitation is that we used only one method to evaluate Eustachian tube function. There are other objective methods for quantifying Eustachian tube function, for instance, tubomanometry and sonotubometry. Using other tests together, the diagnosis of ETD can be more confirmative and can compare the diagnostic efficacies between tests. However, because these tests are unavailable in our clinical setting, we had to use ETDQ7 to support the diagnosis of ETD. Although ETDQ7 showed significant differences between groups, it was a subjective survey. A combination of two or more methods would increase the reliability of future research.

In conclusion, the nine-step test has moderate diagnostic performance for oETD and PET. Both diseases showed decreased ETTmd. The results from the PET group may be attributable to pseudo-dysfunction due to the failure of pressure maintenance.

## Figures and Tables

**Table 1:** Demographic data of enrolled subjects.

	Control	oETD	*p*-Value	PET	*p*-Value
Age (year)	29.0 ± 3.9	42.7 ± 19.5	0.069	41.5 ± 17.8	0.027 *
Male (%)	28.0	36.8	0.560	34.5	0.613
Right side (%)	50.0	42.1	0.787	55.2	0.637
Average ETDQ7	1.1 ± 0.2	3.5 ± 1.2	< 0.001 *	2.8 ± 1.1	< 0.001 *
ETTmd (daPa)	27.9 ± 15.0	10.2 ± 8.4	<0.001 *	15.5 ± 12.9	<0.001 *
Middle ear pressure (daPa)	−9.4 ± 11.5	−146.7 ± 109.0	<0.001 *	−10.3 ± 21.8	>0.999
Number of ears	50	19		29	

oETD: obstructive Eustachian tube dysfunction, PET: Patulous Eustachian tube dysfunction, ETTmd: Maximal difference of peak pressure in the nine-step test, *p*-value: post hoc analysis compared to the control group *: *p* < 0.05.

**Table 2:** Diagnostic values of the modified nine-step test.

Disease	AUC	*p*-Value	Cut-Off	Sensitivity	Specificity
			10 daPa	63.2	92.0
oETD	0.875	<0.001 *	13 daPa	73.7	90.0
			15 daPa	79.0	82.0
			10 daPa	48.3	92.0
PET	0.769	<0.001 *	13 daPa	58.6	90.0
			15 daPa	62.1	82.0

oETD: obstructive Eustachian tube dysfunction, PET: Patulous Eustachian tube dysfunction, AUC: Area under curve, *: *p* < 0.05.

## Data Availability

The data sharing is not available due to ethical problems restricted by the Institutional Review Board.
